# wKinMut: An integrated tool for the analysis and interpretation of mutations in human protein kinases

**DOI:** 10.1186/1471-2105-14-345

**Published:** 2013-11-29

**Authors:** Jose MG Izarzugaza, Miguel Vazquez, Angela del Pozo, Alfonso Valencia

**Affiliations:** 1 Structural Biology and BioComputing Programme, Spanish National Cancer Research Centre (CNIO), C/Melchor Fernandez Almagro, 3, E-28029 Madrid, Spain; 2 Department of Systems Biology, Center for Biological Sequence Analysis, Technical University of Denmark, 2800 Lyngby, Denmark

## Abstract

**Background:**

Protein kinases are involved in relevant physiological functions and a broad number of mutations in this superfamily have been reported in the literature to affect protein function and stability. Unfortunately, the exploration of the consequences on the phenotypes of each individual mutation remains a considerable challenge.

**Results:**

The wKinMut web-server offers direct prediction of the potential pathogenicity of the mutations from a number of methods, including our recently developed prediction method based on the combination of information from a range of diverse sources, including physicochemical properties and functional annotations from FireDB and Swissprot and kinase-specific characteristics such as the membership to specific kinase groups, the annotation with disease-associated GO terms or the occurrence of the mutation in PFAM domains, and the relevance of the residues in determining kinase subfamily specificity from S3Det. This predictor yields interesting results that compare favourably with other methods in the field when applied to protein kinases.

Together with the predictions, wKinMut offers a number of integrated services for the analysis of mutations. These include: the classification of the kinase, information about associations of the kinase with other proteins extracted from iHop, the mapping of the mutations onto PDB structures, pathogenicity records from a number of databases and the classification of mutations in large-scale cancer studies. Importantly, wKinMut is connected with the SNP2L system that extracts mentions of mutations directly from the literature, and therefore increases the possibilities of finding interesting functional information associated to the studied mutations.

**Conclusions:**

wKinMut facilitates the exploration of the information available about individual mutations by integrating prediction approaches with the automatic extraction of information from the literature (text mining) and several state-of-the-art databases.

wKinMut has been used during the last year for the analysis of the consequences of mutations in the context of a number of cancer genome projects, including the recent analysis of Chronic Lymphocytic Leukemia cases and is publicly available at http://wkinmut.bioinfo.cnio.es.

## Background

Current high-throughput resequencing screenings [1-3] represent a powerful set of techniques to discover large numbers of mutations. Of these, only a small fraction are causally implicated in disease onset and therefore, separating the wheat from the chaff is still a major challenge [4]. The interpretation of the overwhelming wealth of data also represents an issue in other fields, such as protein function prediction [5]. For a small subset of the new mutations discovered, experimental information regarding the relationship between the mutation and the underlying biochemical mechanism is known. However, there is no information for the remaining mutations. The intensive requirement of resources makes it unfeasible to experimentally test the association of all these mutations to disease, and to characterize their functional effects. Nevertheless, this problem is very amenable to *in silico* predictors [4,6,7]. Different approaches are currently available to predict the probability of a newly discovered mutation being implicated in disease. Some methods identify crucial positions in a given protein and derive generalized rules to predict the pathogenicity of mutations. Other methods assume that evolutionarily conserved protein residues are important for protein structure, folding and function, whereby mutations in these residues are considered deleterious [8]. Variations on this principle lead to methods that predict deleterious mutations by evaluating changes in evolutionarily conserved PFAM motifs [9]. A number of systems use protein structures to characterize substitutions that significantly destabilize the folded state. There are also methods that integrate prior knowledge in the form of both sequence and structure-related features from a set of experimentally characterized mutations to train automatic machine-learning systems. These systems can infer the pathogenicity of new mutations based on the cases evaluated. Albeit similar in purpose, very different machine-learning methods can be implemented. Among them, probably the most popular ones are: rule-based systems [10-12], decision trees [13], random forests [14,15], neural networks [16,17], Bayesian methods [18] and SVMs [19-23]. Recently, some meta approaches that combine different methodologies have been implemented. For example, Condel [24] integrates five of the most widely employed computational tools for detecting pathogenic single nucleotide variations. Predictors can also be classified according to their scope. Most of the predictors are generally applicable to amino acid sequences from any protein family, while a few of them include properties that apply only to a given protein family of interest; i.e. protein kinase specific predictors [20,23]. These family-related features bring discriminative information that justifies the development of specialized predictors.

A broad number of mutations in the protein kinase superfamily have been reported in the literature [25] and a subset of them is known to disrupt protein structure and function [26]. For some cases, since human protein kinases are involved in a plethora of physiological functions, this disruption can be causally associated to disease [27]. Still, the majority of protein kinase mutations are tolerated without apparent significant effects [28,29].

In previous publications, we have discussed the preferential distribution of germline pathogenic deviations [30] and driver somatic mutations [31] with respect to regions of functional and structural importance. Here we present, wKinMut, an integrated web-service for the collection of information from multiple sources and for the prediction of the pathogenicity of mutations by combining several prediction approaches. The objective of wKinMut is to provide a one-stop resource for the analysis and interpretation of the consequences of mutations in the protein kinase superfamily.

## Implementation

wKinMut represents the first resource to provide an integrated tool for the analysis and interpretation of the consequences of mutations in the protein kinase superfamily. The main objective of wKinMut is to aid computational biologists and clinicians to prioritize pathogenic mutations and to understand the mechanisms by which some mutations lead to disease, and particularly, to cancer.

The tool presented here, incorporates information retrieval and prediction approaches and displays information from diverse sources. First, it simplifies the collection of information about the mutations, such as the classification, domain architecture, functional annotations and plausible interaction partners of the kinase. Furthermore, kinase mutations are analyzed in their structural context and mentions in dedicated databases, genotyping studies and the literature that suggest an implication in disease are also presented. Second, wKinMut estimates the theoretical pathogenicity of kinase mutations with three different approaches, including our newly developed kinase-specific method, KinMut [23], based on the evaluation of a wide set of sequence-derived features that describe each independent mutation. The affected domain and kinase group, diverse functional annotations, residue physicochemical properties and relevance of the mutated residues in determining subfamily specificity are considered.

wKinMut has been implemented mostly in Ruby. The functionality is implemented as a workflow accessible through a REST interface that can render the results either in JSON format or HTML. The later constitutes the interface described in this document. Some of data resources that support this system, such as gene descriptions or iHOP interactions, are queried remotely through the internet as demanded; but are then cached to improve subsequent accesses. The server incorporates some additional caching schemes to improve performance in the back-end, by persisting the job results, and in the web interface, by caching the HTML.

### Web interface

#### Step 1: submission of mutations for analysis

**Figure 1:**
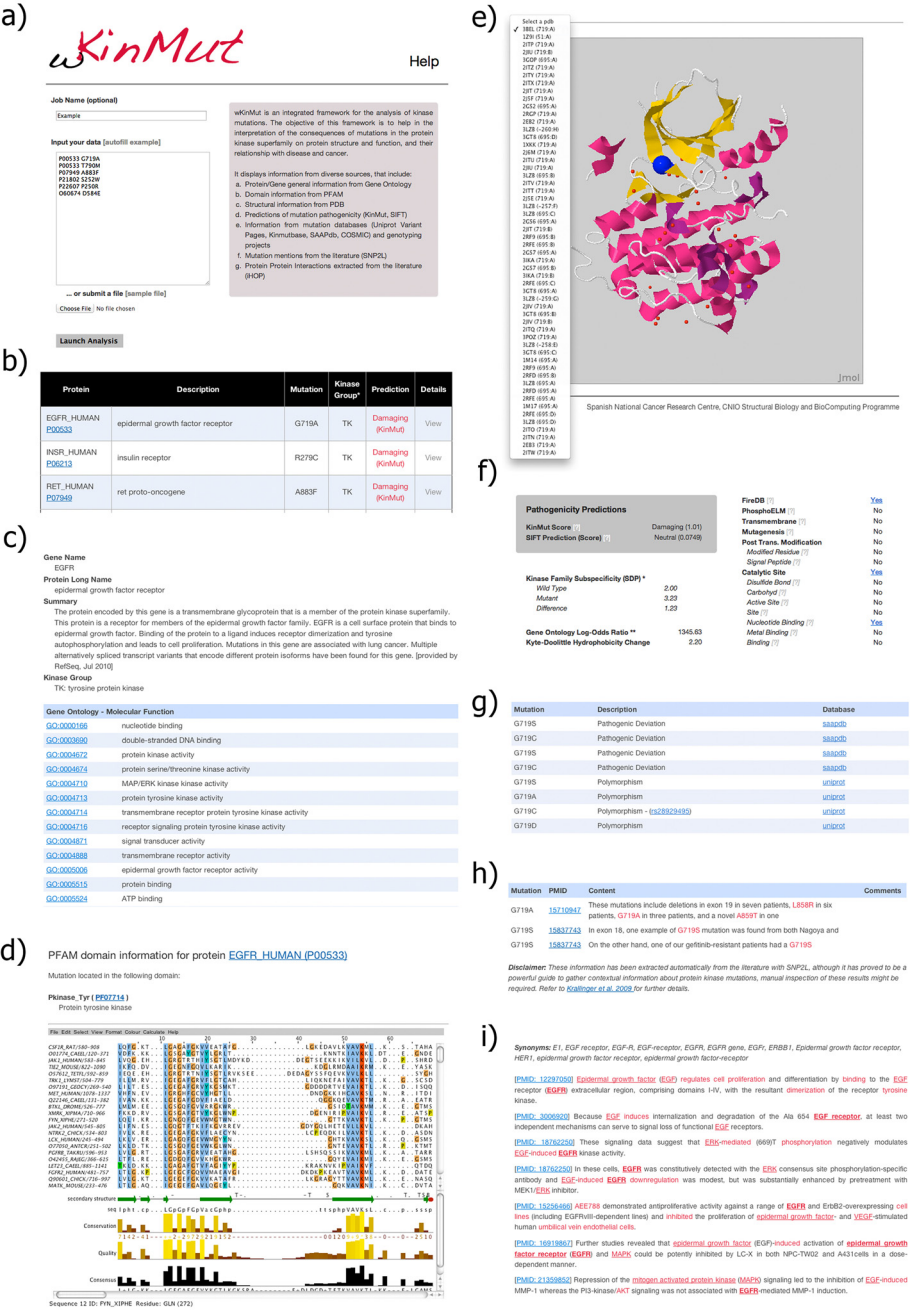
**Summary of the different result pages in wKinMut: Example of a Gly-719-Ala mutation in the human epidermal growth factor receptor.** The figure shows an example of an input to the server (panel **a**) and the results summary table (panel **b**). The rest of panels display show the different outputs from the server, including the gene/protein summary tab (panel **c**), the domain tab (panel **d**), the structure view (panel **e**), the pathogenicity assessment (panel **f**). Information from the databases, the literature and iHop is exemplified in panels **g**, **h** and **i** respectively.

The input to wKinMut are non-synonymous mutations in the protein kinase superfamily. The input format should encode the Uniprot/Swissprot accession number, the wild type residue, the position and the mutated residue. Non-standard amino acids and truncating mutations will be excluded from the analysis. An example of this format would be a mutation from Glycine to Alanine in position 719 of the human epidermal growth factor receptor, which is encoded as P00533 G719A. In the following sections, we will use this example to guide the reader through the different result views (Figure 1). Multiple mutations can be submitted at a time, either as a plain text file or directly via the applications form, the sample dataset provided as part of wKinMut’s documentation can be used as a formatting guide.

#### Step 2: interpretation of the consequences of the mutations

The first output the user will get right after submitting the mutations is a summary page with useful information about the requested mutations (Figure 1, panel b). It includes a description of the proteins in Uniprot, the membership to kinase groups in the classification in KinBase [32,33] and the estimation of the pathogenicity of mutations attending to our kinase-specific predictor of pathogenicity, KinMut [23]. The prediction of the pathogenicity will be discussed in detail in a forthcoming section, nevertheless we decided to include this information at this step as a guide to prioritize mutations. It might be interesting to point out here that users interested only in the results from KinMut, can find a link to the predictions in this summary page that can be accessed programmatically. The scope of wKinMut goes beyond providing raw prediction of pathogenicity from KinMut, the web-service’s main goal is to aid computational biologists and clinicians to understand and to interpret the consequences of kinase mutations. Hence, information complementary to KinMut predictions, is provided. In the summary table, the ‘View’ link in the right-most ‘Details’ column (Figure 1, panel b) will redirect the user to another page containing this complementary information, which includes: the values of the features used for classification, PFAM domains affected by the mutation, protein-protein interaction information extracted from the literature with iHop [34], mentions of the mutations in the literature automatically mined with SNP2L [25,35], and existing records of the mutations in other dedicated databases. This additional information is intended to provide the basic background to help to understand and interpret the consequences of the mutations. Each individual piece of information will be discussed thoroughly in the following sections.

##### General information about the protein/gene

Information under the ‘gene/protein’ tab (Figure 1, panel c) focuses on information shared by all mutations in the same kinase. Background information such as the gene name, the formal description in Uniprot and the classification in KinBase [32,33] of the kinase is provided. In addition, the system provides the Gene Ontology terms with which the kinase has been annotated in each of the independent sub-ontologies (namely Molecular Function, Cellular Compartment and Biological Process). This information provides clues to unveil the function of the kinase and it is used by KinMut to calculate the likeness of the protein (and subsequently the mutation) to play a role in disease.

##### PFAM domains

In a previous publication [23] we demonstrated that mutations occurring in certain domains such as the Tyrosine kinase domain (PKinase Tyr, according to PFAM) are more likely to cause disease. This is coherent with the assumption that the function of some domains is more important than the function of others. In wKinMut, this information is contained in the ‘PFAM domains’ tab (Figure 1, panel d), which displays the domain (or domains, in some cases) where the mutation is occurring and the alignment used by PFAM as seed to generate the domain family. The alignment is evaluated in terms of sequence conservation. Under the assumption that conserved regions have been preserved by evolution, this information can help the user to identify important regions in the structure of the domain.

##### Mapping the mutations onto structures

To understand the consequences of mutations might have in protein stability and function it is sometimes useful to study the mutations in their structural contexts. However, mapping mutations from sequences to structures is not always trivial [36]. Under the ‘Structures‘ tab, wKinMut enables the visualization of the mutation mapped to all available structures. (Figure 1, panel e). In addition, the versatility of the Jmol applet implemented in wKinMut allows advanced users to adapt the visualization to their specific needs.

##### Prediction of the pathogenicity

In wKinMut the theoretical pathogenicity of mutations is assessed by two independent methods, namely SIFT [8] and KinMut [23]. This information is displayed in the ‘Pathogenicity’ tab (Figure 1, panel f). SIFT [8] predicts whether non-synonymous mutations are prone to affect protein function. This prediction is based on the degree of conservation of the residues in sequence alignments derived from closely related sequences. A threshold value of 0.05 is used to determine that mutations are likely to be pathogenic. KinMut [23] is a kinase-specific predictor of the pathogenicity of mutations. It relies in a machine-learning approach (SVM) to evaluate a number of sequence-derived features that describe kinase mutations from different perspectives, including: a) at the gene level, the membership to a Kinbase group and Gene Ontology terms. b) at the domain level, the occurrence of the mutation inside a PFAM domain, and c) at the residue level, several properties including amino acid type, functional annotations from Swissprot and FireDB [37], specificity-determining positions, etc. SVM scores greater than -0.5 indicate that the mutation is very likely pathogenic. The values of these features are also displayed in this section of the web-service to aid to interpret the predictions. Please, refer to the original publications for information on the individual characteristics, capabilities and validation of each predictor.

##### Mutations in databases

The wealth of knowledge provided by current research is usually stored in databases. A number of them store information about mutations from diverse perspectives. In wKinMut (Figure 1, panel g) we collect information from four different sources (namely the Uniprot Variant Pages [38], KinMutBase [39], SAAPdb [26] and COSMIC [40]) in an attempt to cover all aspects of protein kinase mutation. The information displayed includes information about the structural consequences of mutations, experiments associating mutations with a certain disease, or the proof that a mutation has been observed in a cancer sample.

##### Automatic extraction of mutations from the literature

Unfortunately, the databases referred in the previous section do not contain all current knowledge about mutations. Even in the cases where a database record exists, the knowledgebase cannot always store all contextual information. The context is sometimes very important for the correct interpretation of the predictions: experimental conditions, patients’ habits and clinical histories, etcetera. wKinMut provides pointers to mentions of the mutations in the literature under the ‘Literature’ tab (Figure 1, panel h). We extract this information automatically using our in-house text mining approach,SNP2L [25]. In brief, SNP2L is a literature mining pipeline for the automatic extraction and disambiguation of singlepoint mutation mentions from both abstracts as well as full text articles, followed by a sequence validation check to link mutations to their corresponding kinase protein sequences.

##### Automatic determination of interaction partners

wKinMut integrates Protein-Protein Interactions (PPI) gathered from iHOP in the homonymous tab (Figure 1, panel i). Briefly, iHOP is a powerful text mining system to automatically extract protein protein interactions from PubMed abstracts. To relate the interaction information with its context, the sentences including the interaction mentions are also provided.

## Conclusion

wKinMut facilitates the exploration of the information available about individual mutations by integrating prediction approaches with the automatic extraction of information from the literature (text mining) and several currently available databases. wKinMut works as an open accessible web server.

The system offers direct prediction of the potential pathogenicity of the mutations from a number of methods, including our recently developed prediction method based on the combination of information from a range of diverse sources with a machine learning system [23]. The features used by our new prediction system include: general physicochemical properties, annotations of known functional sites from FireDB and Swissprot and kinase-specific characteristics such as membership to a specific group of kinases, annotations of disease associations extracted from GO terms and mapping of PFAM domains, and relevance of the residues for the differences between groups of kinases. In addition to the predictions, wKinMut offers a number of integrated complementary services that help to understand the consequences and the mechanism of the mutations. These services include the classification of the kinase, information about associations of the kinase with other proteins extracted directly extracted from the Medline abstracts, the mutations on the corresponding protein structures, and possible relations with pathogenicity recorded in disease-variation databases and from large-scale cancer studies. An important component of wKinMut is the access to information about the mutations extracted directly from the literature. This information is important for the contextualization of the consequences of the mutations. wKinMut uses our previously developed SNP2L [25], that has been shown to provide a substantial addition to the information provided by public databases and repositories.

In summary, we think that wKinMut constitutes a powerful one-stop shop for the study of the potential pathogenic potential of mutations in protein kinases. As such, wKinMut will be of interest for bioinformaticians and computational biologists that can use the information provided by the server programmatically as part of their own analysis pipelines, and it can be also useful to biologists and clinicians who can browse and explore punctual information easily from the provided interface. We have used wKinMut during the past year for the analysis of the consequences of mutations in the context of a number of personalized cancer genome projects (see [41]), including the recent analysis of Chronic Lymphocytic Leukemia cases [42,43].

A further development of the presented system would consider the analysis of the downstream consequences of mutations in relation to potential and known post-translational modifications and their interelations (see [44,45]). We are interested in extending wKinMut capabilities to the analysis of the combined effect of mutations in pathways and signalling networks in where kinases are essential components wKinMut is publicly available at http://wkinmut.bioinfo.cnio.es.

## Competing interests

The authors declare that they have no competing interests.

## Author’s contributions

AV and JMGI designed the experiment. MV, JMGI designed the web server. MV, JMGI and AP implemented the web server. JMGI, MV and AV wrote the paper. All the authors read and approved the manuscript.
